# Biomarkers for Immune Checkpoint Inhibitor-Mediated Tumor Response and Adverse Events

**DOI:** 10.3389/fmed.2019.00119

**Published:** 2019-05-29

**Authors:** Yoshiyuki Nakamura

**Affiliations:** Department of Dermatology, Faculty of Medicine, University of Tsukuba, Tsukuba, Japan

**Keywords:** immune check point inhibitor, adverse event (AE), PD-1, CTLA- 4, tumor response

## Abstract

In the last decade, inhibitors targeting immune checkpoint molecules such as cytotoxic T-lymphocyte antigen 4 (CTLA-4), programmed cell death 1 (PD-1), and programmed cell death-ligand 1 (PD-L1) brought about a major paradigm shift in cancer treatment. These immune checkpoint inhibitors (ICIs) improved the overall survival of a variety of cancer such as malignant melanoma and non-small lung cancer. In addition, numerous clinical trials for additional indication of ICIs including adjuvant and neo-adjuvant therapies are also currently ongoing. Therefore, more and more patients will receive ICIs in the future. However, despite the improved outcome of the cancer treatment by ICIs, the efficacy remains still limited and tumor regression have not been obtained in many cancer patients. In addition, treatment with ICIs is also associated with substantial toxicities, described as immune-related adverse events (irAEs). Therefore, biomarkers to predict tumor response and occurrence of irAEs by the treatment with ICIs are required to avoid overtreatment of ICIs and minimize irAEs development. Whereas, numerous factors have been reported as potential biomarkers for tumor response to ICIs, factors for predicting irAE have been less reported. In this review, we show recent advances in the understanding of biomarkers for tumor response and occurrence of irAEs in cancer patients treated with ICIs.

## Introduction

The recent development of immune checkpoint inhibitors (ICIs) has led to dramatic advances in cancer therapy. Ipilimumab is a monoclonal antibody to cytotoxic T-lymphocyte antigen 4 (CTLA-4), an inhibitory receptor expressed by both conventional and regulatory T cells (Tregs) and suppresses T cell activation by competing with CD28 to bind CD80/86. Ipilimumab not only activates conventional T cells at the initial stage of maturation but also may show antibody-dependent cell-mediated lysis of the Tregs that play a vital role in suppressing the antitumor immune response (1, 2). Programmed cell death 1 (PD-1) is an inhibitory receptor expressed mainly by activated T cells and its ligand, PD-L1, is widely expressed in cell types as diverse as epithelial cells, immune cells, and cancer cells. Both anti-PD-1 antibodies (nivolumab and pembrolizumab) and anti-PD-L1 antibodies (atezolizumab, durvalumab, and avelumab) exert antitumor effects by activating previously primed T cells which have lost effector and proliferative functions (3). ICIs firstly demonstrated efficacy for patients with advanced melanoma (4–6) and subsequently in other cancers, such as non-small cell lung cancer (NSCLC) and renal cell carcinoma (7–9). A recent clinical trial revealed that adjuvant therapies with anti-PD-1 antibodies prolonged recurrence-free survival in resected high-risk melanoma (10–12). Moreover, there are currently ongoing trials for neoadjuvant therapies with anti-PD-1 antibodies in high risk resectable melanoma (11, 13). Numerous clinical trials testing additional indications of ICIs for other cancers are also ongoing (14, 15). Therefore, an ever-increasing number of patients will receive ICIs in the near future.

However, despite an improved overall survival (OS) with ICIs, the efficacy remains limited and tumor regression has not been universally achieved (16). In addition, use of ICIs may induce unique side effects, described as immune-related adverse events (irAEs). In a previous melanoma phase III clinical trial, patients who received nivolumab alone (*n* = 313), ipilimumab alone (*n* = 311) or nivolumab plus ipilimumab (*n* = 313) saw irAEs of grade 3 or 4 occurring at a rate of 21, 28, and 59%, respectively, and four patients died due to severe irAEs (16). Therefore, biomarkers to predict tumor response and irAE occurrence due to ICIs are necessary to gauge the benefits that each patient will obtain for avoiding overtreatment and minimizing irAEs. Here, we review recent advances in the understanding of biomarkers for tumor response and irAE occurrences.

### Biomarkers for Tumor Response (Table 1)

Numerous factors have been reported as potential biomarkers for objective response rate (ORR), progression free survival (PFS) or OS. However, non-specific factors, which are associated with tumor responses to not only ICIs but also other therapies (such as traditional chemotherapies), can confound the use of these biomarkers. Therefore, specificity as well as correlative strength should be considered in choosing ICIs over other therapies.

**Table 1:** Biomarkers for tumor responses.

**Biomarkers**	**Cancer type**	**Patient number**	**Treatment**	**Key data and clinical significance**	**References**	**Evidence level**
Sex	Melanoma, NSCLC	6,096	Ipilimumab, anti-PD-1 antibodies	PFS and OS of male patients were significantly longer than those of female patients.	Wu et al. (17)	1a
	Melanoma	315	Anti-PD-1 antibodies	Males were significantly associated with better ORR.	Nosrati et al. (18)	2b
Age				Ages older than 65 years correlated with better ORR.		
	Melanoma, prostate cancer, NSCLC, RCC	5,265	Anti-CTLA-4 antibodies, anti-PD-1 antibodies	Ages younger than 75 years correlated with better ORR.	Nishijima et al. (19)	1a
Tumor size	Melanoma	459	Pembrolizumab	Tumor size was independently associated with OS, suggesting that early detection of metastatic lesions may be important for better response to ICIs.	Joseph et al. (20)	2b
TILs	Melanoma	46	Pembrolizumab	High density of CD8^+^ TILs at the invasive margin correlated with better tumor response. An increase in CD8^+^ TILs from baseline to post-treatment was associated with tumor regression.	Tumeh et al. (21)	2b
PD-L1 expression in tumors	Melanoma	277	Nivolumab after treatment with anti-CTLA-4 antibodies	Better ORR were observed in patients with positive PD-L1 expression in tumors.	Weber et al. (22)	1b
	Melanoma	451	Pembrolizumab	Better PFS and OS were observed in patients with positive PD-L1 expression in tumors.	Daud et al. (23)	2b
	NSCLC	410	Pembrolizumab + chemotherapy	Higher PD-L1 expression was associated with better PFS and OS.	Gandhi et al. (24)	1b
ICOS	Melanoma	14	Ipilimumab	Increased expression of ICOS on CD4^+^ T cells that is sustained for more than 12 weeks correlated with improved OS.	Carthon et al. (25)	4
TIM-3	Melanoma	67	Ipilimumab	Increased TIM-3 expression on circulating T and NK cells prior to and during treatment was associated with shorter OS.	Tallerico et al. (26)	2b
IDO	Melanoma	82	Ipilimumab	Baseline IDO expression in tumor tissue assessed by IHC correlated with better ORR.	Hamid et al. (27)	1b
	NSCLC	26	Nivolumab	IDO activity as assessed by serum kynurenine/tryptophan ratio was negatively associated with longer PFS and OS.	Botticelli et al. (28)	2b
Soluble CTLA-4	Melanoma	113	Ipilimumab	Higher serum levels of soluble CTLA-4 at baseline had both better ORR and OS.	Pistillo et al. (29)	2b
Soluble PD-L1	Melanoma	446	Ipilimumab, anti-PD-1 antibodies	Higher levels of baseline soluble PD-L1 were associated with worse response. Increases in soluble PD-1 after treatment was associated with favorable clinical responses.	Zhou et al. (30)	2b
	NSCLC	39	Nivolumab	Higher levels of baseline soluble PD-L1 were associated with shorter OS.	Okuma et al. (31)	2b
Soluble CD163	Melanoma	59	Nivolumab	Serum levels of soluble CD163 were increased after 6 weeks in responders compared to non-responders after initial treatment for cutaneous melanoma.	Fujimura et al. (32)	2b
Soluble NKG2D	Melanoma	194	Anti-CTLA-4 antibodies, anti-PD-1 antibodies	Higher levels of circulating soluble ULBP-1, soluble ULBP-2 and LDH at baseline were independent factors of shorter OS.	Maccalli et al. (33)	2b
IFN-γ	Melanoma	45	Ipilimumab	The post-treatment expression levels of IFN-γ responsive genes in tumor tissues were associated with longer OS.	Ji et al. (34)	2b
	NSCLC	97	Durvalumab	High levels of pre-treatment IFN-γ expression and its related genes in tumor tissues were associated with longer OS.	Higgs et al. (35)	2b
	Melanoma	43	Atezolizumab	High expression of IFN-γ and CXCL-9 was associated with better ORR.	Herbst et al. (36)	2b
TNF-α	Melanoma	15	Nivolumab	Patients who showed complete remission, partial remission or long-term stable disease due to nivolumab response had lower serum levels of TNF-α compared to non-responders.	Tanaka et al (37)	4
Lymphocyte counts	Melanoma	209	Ipilimumab	Higher levels of relative lymphocyte counts at baseline were associated with longer OS.	Martens et al. (38)	2b
	Melanoma	50	Ipilimumab	Absolute lymphocyte counts after treatment were associated with longer OS.	Wilgenhof et al. (39)	2b
	Melanoma	98	Nivolumab	Absolute lymphocyte counts after treatment correlated with better OS.	Nakamura et al. (40)	2b
Eosinophil counts	Melanoma	209	Ipilimumab	High absolute and relative eosinophil counts at baseline were associated with a longer OS.	Martens et al. (38)	2b
	Melanoma	616	Pembrolizumab	Relative eosinophil counts at baseline were an independent factor for longer OS and better ORR.	Weide et al. (41)	2b
	Melanoma	59	Ipilimumab	Early increases in absolute eosinophil counts from baseline during treatment were an independent factor for better responses.	Gebhardt et al. (42)	2b
NLR	Melanoma	90	Nivolumab	NLR was associated with poor tumor response.	Fujisawa et al. (43)	2b
	NSCLC	175	Nivolumab	NLR was associated with poor tumor response.	Bagley et al. (44)	2b
	Melanoma	44	Anti-PD-1 antibodies	NLR was the only factor associated with both poor ORR and shorter PFS.	Nakmaura et al. (45)	2b
Tregs	Melanoma	209	Ipilimumab	High levels of circulating Tregs at baseline were associated with longer OS.	Martens et al. (38)	2b
	Melanoma	95	Ipilimumab	Decreasing levels of circulating Tregs were associated with better responses.	Simeone et al. (46)	2b
MDSC	Melanoma	92	Ipilimumab	The baseline frequency of MDSCs in blood correlated with shorter OS.	Weber et al. (47)	2b
	Melanoma	83	Nivolumab	The baseline frequency of MDSCs in blood correlated with shorter OS.	Kitano et al. (48)	2b
	Prostate cancer	28	Ipilimumab plus a cancer vaccine	The baseline frequency of circulating MDSCs correlated with shorter OS.	Santegoets et al. (49)	2b
LDH	Melanoma	73	Ipilimumab	High baseline LDH was associated with poor anti-tumor response.	Delyon et al. (50)	2b
CRP	Melanoma	95	Ipilimumab	A decrease or no change in serum levels of CRP from baseline was associated with longer OS.	Simeone et al. (46)	2b
Mutation burden	Melanoma	64	Ipilimumab	High mutation burden was associated with a longer OS.	Synder et al. (51)	2b
	Melanoma	150	Ipilimumab	High mutation burden was associated with tumor responses.	Allen et al. (52)	2b
MSI	Colorectal cancer	74	Nivolumab	A high response to anti-PD-1 antibodies in colorectal cancer with high levels of MSI compared to traditional treatments was observed.	Overman et al. (53)	2b
HLA	Melanoma	13	Nivolumab	HLA-A expression in pre-treatment was elevated in responders compared to non-responders.	Inoue et al. (54)	4
	Melanoma	69	Nivolumab	HLA-A26 correlated with tumor response to nivolumab in Japanese melanoma patients.	Ishida et al. (55)	2b
T cell repertoire	Melanoma	12	Ipilimumab	Both higher richness and evenness in pre-treatment peripheral blood were associated with a better response.	Postow et al. (56)	4
	Melanoma	46	Pembrolizumab	TILs with less diversity were associated with clinical response.	Tumeh et al. (21)	2b
Gut microbiome	Melanoma	26	Ipilimumab	Patients whose baseline microbiota was enriched with *Faecalibacterium* genus and other Firmicutes showed a longer PFS and OS than those whose baseline microbiota was enriched with *Bacteroides*.	Chaput et al. (57)	2b
	Melanoma	43	Anti-PD-1 antibodies	A higher diversity of gut microbiome and relative abundance of *Ruminococcaceae* family bacteria correlated with better ORR and longer PFS.	Gopalakrishnan et al. (58)	2b
	NSCLC, RCC	100	Anti-PD-1 antibodies	The relative abundance of *Akkermansia muciniphilia* was associated with better responses.	Routy et al. (59)	2b
ctDNA	Melanoma	76	Anti-PD-1 antibodies	Patients with a persistently elevated cDNA during the treatment showed a worse response and shorter PFS and OS. ctDNA may be a useful marker for differentiating pseudoprogression from true progression during immune checkpoint inhibitor treatment.	Lee et al. (60, 61)	2b
Exosomal molecules	Melanoma	44	Pembrolizumab	Lower baseline levels and increases during the treatment in exosomal PD-L1 protein correlated with tumor response.	Chen et al. (62)	2b
	Melanoma, NSCLC	26	Anti-PD-1 antibodies	Baseline exosomal PD-L1 mRNA expression was higher in responders, and exosomal PD-L1 mRNA expression in responders was decreased after treatment whereas it was stable in stabilized patients and increased in progressive disease cases.	Re et al. (63)	2b
	Melanoma	59	Ipilimumab	Increased exosomal PD-1 and CD28 levels in T cells were associated with longer PFS and OS while increased exosomal CD80 and CD86 in dendritic cells correlated with longer PFS.	Tucci et al. (64)	2b
irAE development	RCC	40	Ipilimumab	Overall irAEs were associated with tumor responses.	Yang et al. (65)	2b
	NSCLC	43	Nivolumab	Early development of all irAEs was associated with better ORR and longer PFS.	Teraoka et al. (66)	2b
	NSCLC, RCC, HNSCC, urothelial carcinoma	142	Anti-PD-1 antibodies	Only low grade irAEs were associated with better responses.	Judo et al. (67)	2b
	Melanoma	60	Ipilimumab after nivolumab	Occurrences of endocrine irAEs were associated with longer OS.	Fujisawa et al. (68)	2b
	Melanoma	5,737	Anti-CTLA-4 antibodies, anti-PD-1 antibodies	Development of vitiligo correlated with better responses.	Teuling et al. (69)	2a

*NSCLC, non-small cell lung cancer; RCC, renal cell carcinoma; HNSCC, head and neck squamous cell carcinoma; ORR, overall response rate; PFS, progression free survival; OS, overall survival; TILs, tumor infiltrating lymphocytes; ICOS, inducible T cell costimulatory; IDO, indoleamine 2,3-dioxygenase; NLR, neutrophil-to-lymphocyte ratio; Tregs, regulatory T cells; MDSC, myeloid-derived suppressor cells; MSI, microsatellite instability; HLA, human leukocyte antigen; ctDNA, circulating tumor DNA; irAE, immune-related adverse event. Evidence level was evaluated based on the following criteria; 1a, systematic review/ meta-analysis of randomized controlled trials; 1b, individual randomized controlled trials; 2a, systematic review/ meta-analysis of cohort studies; 2b, individual cohort study; 3a, systematic review/meta-analysis of case-control studies; 3b, individual case-control studies; 4, case series; 5, expert opinions*.

### Sex

Several studies have demonstrated that sex differences are associated with anti-tumor immune responses (70, 71). Although many clinical studies did not show a correlation between sex and tumor response to ICIs, meta-analyses with larger numbers of melanoma and NSCLC patients who were treated with ICIs revealed that both the PFS and OS of male patients were significantly longer than those of female patients (17). Based on a subtype analysis, sex differences in OS were greater in melanoma patients than NSCLC patients. In addition, in the anti-CTLA-4 antibodies group, the OS difference between male and female was greater than in the anti-PD-1 antibodies group. In line with this result, another study demonstrated that males were significantly associated with better ORR in melanoma patients treated with anti-PD-1 antibodies (18). Therefore, males seem to benefit more from ICIs than females do although the mechanism behind this effect has yet to be clarified.

### Age

A recent preclinical study demonstrated that tumor response to anti-PD-1 antibodies in aged mice was significantly increased compared to younger mice, an effect attributed to the lower proportion of Tregs in aged mice (72). Consistent with these results, the tumor response to pembrolizumab in melanoma patients over age 60 was significantly higher than those under 60 years and the likelihood of response increased with age (72). Similarly, Nosrati et al showed that ages older than 65 years correlated with better ORR in melanoma patients treated with anti-PD-1 antibodies (18). However, opposite results have also been reported and a meta-analysis by Nishijima et al revealed a correlation between ages younger than 75 years with better ORR in patients treated with ICIs (19). Therefore, further studies are needed to evaluate the usefulness of age as a biomarker for ICI response.

### Tumor Size

Huang et al. reported that reinvigoration of exhausted CD8 T cells (T_ex_-cell) positively correlated with tumor size and the ratio of T_ex_-cell reinvigoration to tumor size was significantly associated with better ORR and longer OS in melanoma patients treated with pembrolizumab (73), indicating that tumor size is a predictive factor for poor response to ICI treatments. Indeed, another study demonstrated that tumor size was independently associated with OS in melanoma patients treated with pembrolizumab although it was associated with many other clinical factors (20). Therefore, early detection of metastatic lesions may be important for better response to ICIs.

### Immune Cell Infiltration

Because ICIs activate the immune response to cancer, infiltration of immune cells, including T cells, into tumors may induce tumor regression following treatment. Generally, higher numbers of tumor infiltrating lymphocytes (TILs) have been a favorable prognostic factor in many types of cancers, such as melanoma and colorectal cancer (74, 75). Similarly, Tumeh et al revealed that presence of CD8^+^ TILs at the invasive margin, which was associated with higher PD-1/PD-L1 expression, correlated with better tumor response in melanoma patients treated with pembrolizumab (21). An increase in CD8^+^ TILs from baseline to post-treatment biopsy, specifically at the tumor center and invasive margin, has been also significantly associated with tumor regression (21). Therefore, both baseline and post-treatment TIL numbers may be important biomarkers for predicting tumor response to ICIs.

### Surface Molecules and Their Related Molecules

#### PD-L1

Since PD-L1 is a ligand of PD-1 and serves an inhibitory signal in PD-1 expressing cells, the expression of PD-L1 in tumor environments is speculated to correlate with better response in patients treated with anti-PD-1 antibodies. Indeed, in melanoma clinical trials with anti-PD-1 antibodies, better outcomes were observed in patients with positive PD-L1 expression in tumors although the definition of positive or negative expression differed across studies (22, 23). Higher PD-L1 expression has also been associated with better outcomes in NSCLC patients treated with anti-PD-1 antibodies (24). In addition, a recent clinical trial demonstrated that combinations of nivolumab with ipilimumab showed a better OS than nivolumab monotherapy in melanoma patients with PD-L1 <1%, whereas the OS was comparable between the 2 treatment groups in patients with PD-L1≥1%, suggesting that anti-PD-1 antibody efficacy is largely dependent on PD-L1 expression (16). Therefore, PD-L1 expression may be a vital factor to predict tumor response to anti-PD-1 antibodies although tumor responses can be also observed in PD-L1 negative tumors. However, issues remain for accurately assessing PD-L1 expression, including different antibodies used in each study, and the low reproducibility of pathologist evaluations (76). In addition, PD-L1 expression has been reported to vary between primary tumors and metastatic sites (77). Therefore, establishing evaluative standards for tumor PD-L1 expression will enhance its usefulness as a predictive factor.

### Inducible T Cell Co-stimulator (ICOS)

ICOS is a co-stimulating molecule expressed by activated conventional T cells and regulatory T cells. A previous report demonstrated that ipilimumab treatment increases expression of ICOS on conventional CD4^+^ T cells in both blood and tumor tissue in patients with bladder cancer (78). These CD4^+^ ICOS^+^ T cells produced IFNγ and could recognize tumor antigens (78). In addition, increased expression of ICOS on CD4^+^ T cells that is sustained for more than 12 weeks has been reported to correlate with improved survival in melanoma patients treated with ipilimumab (25). Thus, ICOS expression is a potential biomarker for tumor response to ICIs although further studies are needed to establish its utility.

### Other Cell Surface Molecules

Pre-clinical studies using mouse models have indicated that upregulation of alternative inhibitory molecules causes resistance to anti-PD-1 antibody therapy (79). These molecules include TIM-3, LAG-3, and VISTA are therefore suggested to serve as potential target molecules for alternative checkpoint inhibitors. They could also serve as potential biomarkers for ICI response and, indeed, increased TIM-3 expression on circulating T and NK cells prior to and during treatment has been significantly associated with shorter OS in melanoma patients treated with ipilimumab (26).

### Enzymes Related to Immune Response

#### Indoleamine 2,3-dioxygenase (IDO)

IDO is an enzyme that converts the essential amino acid l-tryptophan into kynurenine and carries an immunosuppressive effect through multiple mechanisms (80). Kynurenine, mediated by IDO, has been shown to induce T cell apoptosis (81), and IDO-induced starvation of tryptophan mediates the conversion of naïve CD4^+^ T cells into Tregs through GCN2 kinase activation (82). A recent study demonstrated that IDO expression levels in melanoma cells were independently associated with tumor stage (83). IDO has been also reported as a predictor of anti-tumor response by ICIs. Hamid et al showed that baseline IDO expression, as well as baseline FoxP3 expression, in tumor tissue assessed by IHC significantly correlated with better ORR in melanoma patients treated with ipilimumab (27). However, on the contrary, IDO activity as assessed by serum kynurenine/tryptophan ratio has been negatively associated with longer PFS and OS in NSCLC patients treated with nivolumab (28). Therefore, through as-yet unknown mechanisms, IDO activity may serve as a predictive marker for outcomes, which are different dependent on the assessment.

### Soluble Isoform of Surface Molecules

#### Soluble CTLA-4 (sCTLA-4)

Soluble CTLA-4 originates from a spliced variant of an alternative transcript that lacks the transmembrane sequence (84). It can be detected in normal human serum and higher levels of sCTLA-4 have been observed in autoimmune diseases and many types of cancers (84, 85). It can bind to CD80/86 on antigen-presenting cells and block the binding of membrane-bound CTLA-4 or CD28 on T cells, thus avoiding the downregulation of the immune activation cascade (86, 87). Pistill et al. demonstrated that higher serum levels of sCTLA-4 (>200 pg/ml) at baseline had both better ORR and OS than lower sCTLA-4 serum levels (≤200 pg/ml) in melanoma patients treated with ipilimumab (29), suggesting that serum sCTLA-4 could be a biomarker for better response to ipilimumab. It is speculated that sCTLA-4 might block the binding of membrane-bound CTLA-4 to its ligand and thus result in enhanced tumor immunity in synergy with ipilimumab.

### Soluble PD-L1 (sPD-L1)

Soluble PD-L1 may result from alternative variants of the PD-L1 transcripts and cytokine treatment with IFN-α, IFN-γ, or TNF-α has been shown to increase secretion of sPD-L1 as well as expression of cell surface PD-L1 in melanoma cell lines (30). It can be detected in blood and elevated levels of circulating sPD-L1 have been associated with poor prognoses in many types of cancer (88–90). Consistent with these results, higher levels of baseline sPD-L1 have been significantly associated with worse response and shorter OS in melanoma patients treated with ICIs (30, 31). Therefore, baseline sPD-L1 could represent an immune suppressive state and poor response to ICIs although the function of sPD-L1 is not fully understood. In contrast, increases in sPD-1 after treatment with ICIs have been associated with favorable clinical responses (30). Secretion of sPD-1 after ICI treatment may be caused, at least partially, by enhanced production of cytokines such as IFN-α, IFN-γ, or TNF-α due to ICI-mediated anti-tumor response because altered levels of sPD-1 after treatment of ipilimumab corresponded to changes in the circulating cytokines (30).

### Soluble CD163 (sCD163)

CD163 is a member of the scavenger receptor family and is mainly expressed by macrophages/monocytes (91). Several reports have shown that CD163^+^ M2 macrophages comprised the main population of the tumor-associated macrophages (TAMs) that play important roles for suppressing anti-tumor immune responses and serum levels of sCD163, generated by proteolytic shedding, is thought to be a marker for TAMs (91, 92). Fujimura et al. reported that serum levels of sCD163 were significantly increased after 6 weeks in responders compared to non-responders after initial treatment with nivolumab for cutaneous melanoma (32). Interestingly, such an increase was not observed in patients with mucosal melanoma although the mechanism for this phenomenon remains unclear. These results suggest that sCD163 may serve as a biomarker for patients with specific types of cancer treated with ICIs.

### Soluble NKG2D Ligands (sNKG2DLs)

NKG2D is a member of the C-type lectin-like receptors and is expressed on T, NK, and NKT cells (93, 94). The binding of NKG2D with its ligands [MHC class I chain-related gene [MIC] and UL-16-binding protein [ULBP]] elicits activation signals to NK and T cells (93–95). NKG2DLs are usually absent on the surface of normal cells but are induced by various stressors (such as DNA damage) and are often overexpressed by cancer cells (93, 95, 96). Soluble NKG2DLs, generated as result of proteolytic shedding by tumor cells, can be detected in serum and their levels have been reported to correlate with tumor progression (94, 95, 97, 98). Soluble NKG2DLs suppress anti-tumor immune responses through multiple mechanisms that include the binding and subsequent endocytosis and degradation of NKG2D on NK and T cells (97, 99, 100). A multivariate analysis conducted by Maccalli et al. showed that higher levels of circulating sULBP-1, sULBP-2, and LDH at baseline were independent factors of shorter OS in melanoma patients treated with ICIs (33). Interestingly, only LDH, but not sNKG2DLs, significantly correlated with outcomes in patients treated with other therapies, such as chemotherapies and BRAF inhibitors (33), suggesting that soluble circulating sULBP-1 and sULBP-2 may be indicators that use of ICIs is more suitable than other therapies.

### Cytokines and Chemokines

#### IFN-γ

IFN-γ is a functionally pleiotropic cytokine that modulates the expression of numerous proteins in exposed cells (101). PD-L1 expression is also upregulated mainly controlled by IFN-γ (102). IFN-γ and its targets play crucial roles in eliminating tumor cells through direct induction of cytotoxic activities as well as enhancing the Th1-related immune response (101). The post-treatment expression levels of IFN-γ responsive genes in tumor tissues were associated with better outcomes in patients treated with ipilimumab (34). Similarly, high levels of pre-treatment IFN-γ expression and its related genes in tumor tissues are associated with longer OS in NSCLC patients treated with durvalumab (35). Similar associations between high expression of IFN-γ and CXCL-9, an IFN-γ related chemokine, with better ORR was observed in melanoma patients treated with atezolizumab (36). Therefore, high expression of IFN-γ and its associated molecules in tumor tissues may be useful biomarkers that are indicative of a better anti-tumor response to ICIs.

### TNF-α

TNF-α is an inflammatory cytokine produced by various cells, including immune cells and epithelial cells. It promotes tumor growth and higher serum levels of TNF-α have been reported to be associated with poor prognoses in cancer patients (103, 104). Tanaka et al. reported that melanoma patients who showed complete remission, partial remission or long-term stable disease due to nivolumab response had significantly lower serum levels of TNF-α compared to non-responders (37).

### IL-6

IL-6 is produced by a broad variety of cells, including immune cell and tumor cells. It promotes tumor progression via inhibition of cancer cell apoptosis as well as promotion of angiogenesis (105). In a previous study, higher serum IL-6 was associated with shorter OS in melanoma patients treated with IL-2-based immunotherapy (106). Although association of serum IL-6 with response to ICIs has yet to be shown, CRP, whose production is mainly controlled by IL-6 (107), has been reported to be predictive of outcomes in patients treated with ICIs, which bolsters the argument of IL-6 as a potential biomarker of anti-tumor response during ICI treatment.

### Blood Cell Counts

#### Lymphocyte Counts

Because both CTLA-4 and PD-1 are expressed mainly on lymphocytes, several reports have pointed out the association between blood lymphocyte count and tumor response to ICIs (38–40). Martens et al showed that higher levels of relative lymphocyte counts at baseline were significantly associated with longer OS in melanoma patients treated with ipilimumab (38). In another study, absolute lymphocyte counts after 2 doses of ipilimumab were associated with longer OS in melanoma patients (39). Similarly, Nakamura et al showed that absolute lymphocyte counts at week 3 and 6 after the initial administration of nivolumab significantly correlated with better OS in melanoma patients (40). These results suggest that lymphocyte counts both at baseline and after treatment with ICIs may be useful for predicting better outcomes.

### Eosinophil Counts

Eosinophils also play a crucial role in tumor destruction and recruitment of T cells into the tumor environment (108). Indeed, mice with peripheral blood eosinophilia showed substantial tumor suppression (109). In addition, multiple studies have revealed a positive correlation between increased eosinophil infiltration into tumor tissues and a favorable prognosis in many cancers (110, 111). Consistent with this idea, numerous previous studies have reported that higher blood eosinophil counts correlate with favorable outcomes in patients treated with ICIs. Marten et al. demonstrated that in melanoma patients treated with ipilimumab, high absolute, and relative eosinophil counts at baseline were associated with a longer OS (38). Similarly, a multivariate study by Weide et al. demonstrated that relative eosinophil counts at baseline were an independent factor for longer OS and better ORR in melanoma patients treated with pembrolizumab (41). In addition, Gebhardt et al. reported that early increases in absolute eosinophil counts from baseline during ipilimumab treatment were an independent factor for better responses in melanoma patients (42). Therefore, eosinophil counts at both baseline and after ICI treatment may serve as biomarkers for better tumor response.

### Neutrophil-to-Lymphocyte Ratio (NLR)

Fujisawa et al. showed that baseline NLR was associated with poor tumor response in melanoma patients treated with nivolumab (43). Similar findings have also been reported in melanoma patients treated with ipilimumab and NSLC patients treated with nivolumab (41, 44). In a previously published study, our multivariate analysis revealed that NLR was the only factor associated with both poor ORR and shorter PFS in melanoma patients treated with anti-PD-1 antibodies, suggesting that NLR is a strong predictive factor for poor outcome in patients treated with ICIs (45). Given that lymphocytes play vital roles in the ICI-induced immune response to tumors while neutrophilia represents the response to systemic inflammation (112), a high NLR might represent an impaired specific immune response to tumors. However, increased turnover of tumor cells causes the release of large amounts of damage-associated molecular patterns (DAMPs) from tumor debris, leading to recruitment and activation of neutrophils (113, 114). Moreover, numerous reports have also shown that NLR serves as a biomarker for poor response to other treatments, such as chemotherapies and radiation (115, 116). Therefore, the NLR might simply represent rapidly expanding tumor cell populations rather than any potential immune response mediated by ICIs.

### Tregs

Tregs, a population characterized by FoxP3^+^ CD25^+^ CD4^+^ T cells, significantly suppress immune responses (117), and it has been shown that their depletion effectively eradicates tumor cells via an enhanced anti-tumor immune response (118, 119). In addition to their immune suppressive function, they may be a target for antibody dependent cellular cytotoxicity (ADCC) by ipilimumab due to their high expression levels of CTLA-4 that make Tregs sentinels for ICI-mediated anti-tumor responses. Indeed, high levels of circulating Tregs at baseline have been associated with longer OS in melanoma patients treated with ipilimumab (38). In addition, decreasing or stable levels of circulating Tregs 12 weeks after initial administration of ipilimumab significantly correlated with better disease control and longer OS than increasing Treg levels. Furthermore, similar results have been obtained in another study, with decreasing levels of circulating Tregs significantly associated with better responses to ipilimumab (46). Therefore, circulating Tregs both at baseline and after treatment with ipilimumab may be useful biomarkers for anti-tumor response.

### Myeloid-Derived Suppressor Cells (MDSCs)

MDSCs are a heterogeneous population of myeloid origin characterized by a failure to differentiate into granulocytes, macrophages or dendritic cells (120). They expand in tumor environments and strongly suppress the activity of immune cells, including T cells, through a variety of mechanisms such as NO production and arginase-1 overexpression. Both of these processes lead to cell cycle arrest and downregulation of the T cell receptor (120). MDSCs are defined as Lin^−^CD14^+^HLA^−^DR^−/low^ (120) and clinical and experimental studies have shown that high infiltration of these cells into tumor tissues are associated with poor prognosis and resistance to therapies (121, 122). MDSCs can also be detected in the blood and several studies have demonstrated that the baseline frequency of MDSCs in blood significantly correlates with shorter OS in melanoma patients treated with ipilimumab or nivolumab (47, 48). Furthermore, in prostate cancer patients treated with ipilimumab plus a cancer vaccine, the baseline frequency of circulating MDSCs correlated with a shorter OS (49). These results suggest that the frequency of blood MDSCs also serves as a useful biomarker for ICI response.

### Serum Markers

#### Lactate Dehydrogenase (LDH)

Generally, baseline serum LDH is an independent factor for poor prognosis in patients with advanced melanoma (123). The same applies to cases of ICI treatment and numerous reports have demonstrated that high baseline LDH was associated with poor anti-tumor response in various cancer patients who received ICI treatment (50, 124, 125). This poor outcome may simply be caused by increased turnover of tumor cells which enhances LDH release in similar fashion to a high NLR.

### CRP

CRP is produced by hepatocytes and serum levels of it elevate quickly in response to most inflammation (such as bacterial infections). However, CRP does not usually increase during ICI-mediated tumor regression. Simeone et al. reported that a decrease or no change in serum levels of CRP from baseline were significantly associated with longer OS in melanoma patients treated with ipilimumab (46). Therefore, elevated CRP from baseline may indicate inflammation by tumor progression or irAE rather than an antitumor immune response from ICI treatment.

### Genomic Mutations

#### Mutation Burden

Mutation burden, the number of mutations within a tumor genome, is different among and within the cancer types (126). Overall, multiple studies have shown that a high mutation burden was associated with a better response to ICIs (51, 52). This mechanism is not fully understood but an increased number of neoantigens (potential tumor-specific T cell targets) generated by a high mutation burden is thought to cause an enhanced response to ICIs (127). As for melanoma, our study demonstrated that acral lentiginous melanoma (ALM) and mucosal melanoma (MCM), both common types of melanoma in Asians, were less susceptible to immune checkpoint inhibitors than superficial spreading melanoma (SSM) and lentigo maligna melanoma, both major types of Caucasian melanoma (128). This may be explained, at least in part, by the lower mutation burden in ALM and MCM (129). Despite the poor ICI-mediated antitumor response in ALM patients, our retrospective study demonstrated that use of ICIs significantly improved OS in not only SSM but also ALM patients (128).

### Microsatellite Instability

Mutation or silencing of mismatch repair genes, which causes deficient mismatch repair (dMMR), leads to accumulation of multiple mutations and microsatellite instability (MSI). Zhang et al. reported that the immune microenvironment in colorectal cancer differs between dMMR tumors and proficient mismatch repair (pMMR) tumors (130). The number of CD8^+^ TIL, PD-1^+^ TIL and IDO^+^ tumor cells was increased in tumors with dMMR compared to those with pMMR, suggesting that dMMR is indicative of exhausted T-cell-rich environments (130). It has been reported that colon cancer with dMMR frequently shows larger tumors with poorer differentiation (131). In addition, previous studies revealed that patients with dMMR had both a poorer response to conventional chemotherapies and shorter OS than patients with pMMR in many types of cancer (132, 133). However, due to the high mutation burden, several clinical trials revealed a high response to anti-PD-1 antibodies in colorectal cancer with dMMR or high levels of MSI (MSI-H) compared to traditional treatments (53), suggesting that dMMR serves as useful indicator for choosing ICIs over other therapies. Recently, a durable response was observed in patients with dMMR or MSI-H across five clinical trials treated with pembrolizmab (KEYNOTE-016, 164, 012, 028, 158). The cancer types included colorectal, endometrial, biliary, gastric, esophageal, pancreatic and breast cancers. Based on these results, the United States Food and Drug Administration approved pembrolizumab for the treatment of any unresetable or metastatic solid tumors that display dMMR or MSI-H. A combination of nivolumab with ipilimumab was also shown to effect a promising response to dMMR/ MSI-H colorectal cancer (134).

### Human Leukocyte Antigen (HLA)

HLA encodes cell surface molecules which present antigenic peptides to the T-cell receptor (TCR) on T cells. Inoue et al reported that mRNA expression of HLA-A in pre-treatment melanoma was elevated in responders to nivolumab compared to non-responders (54). There are numerous variant alleles at the HLA loci which differ in each individual and Ishida et al reported that HLA-A26, which is relatively common in Japanese but rare in Caucasians, correlated with tumor response to nivolumab in Japanese melanoma patients (55).

### T Cell Receptor (TCR) Repertoire

Since the TCR determines T cell specificity with respect to tumor cells, the TCR repertoire may be predictive of the ICI-induced anti-tumor immune response. As diversity of the repertoire is increased, the likelihood of a specific immune response to tumor cells is speculated to be elevated (56). A previous study showed that both higher richness and evenness in pre-treatment peripheral blood are associated with a better response to ipilimumab in melanoma patients (56). On the other hand, Tumeh et al. showed that TILs with less diversity were significantly associated with clinical response to pembrolizumab in melanoma patients (21). It is speculated that TILs with less diversity contain a higher proportion of tumor-specific T cells, and therefore, the anti-tumor response was enhanced by ICIs. In this study, a TIL clone population expanded more than 10 times in responders than non-responders after treatment with pembrolizumab (21), revealing that both diversity and clonal expansion of T cells may predict ICI response although this indication may differ between blood and tumor tissues.

### Gut Microbiome

Emergent evidence has suggested that the gut microbiome plays crucial roles for the immune response of not only intestinal diseases but also other disorders, including various type of cancers (135). Sivan et al. reported that, in mice, commensal Bifidobacterium enhanced the response to anti-PD-1 antibodies through an augmented dendritic cell function (136). Several studies have also demonstrated that distinct gut microbiota were associated with ICI response in humans. Melanoma patients whose baseline microbiota was enriched with *Faecalibacterium* genus and other Firmicutes showed a longer PFS and OS than those whose baseline microbiota was enriched with *Bacteroides* upon ipilimumab treatment (57). In addition, Gopalakrishnan et al. reported that a higher diversity of gut microbiome and relative abundance of *Ruminococcaceae* family bacteria before starting anti-PD-1 antibodies in melanoma patients correlated with better ORR and longer PFS (58). Moreover, Routy et al. showed that dysbiosis by administration of antibiotics inhibited ICI response in both mice and humans (59). This study also revealed a correlation between clinical responses and the relative abundance of *Akkermansia muciniphilia*. They also showed that transplantation of *Akkermansia muciniphilia* into mice enhanced the efficacy of PD-1 antibodies in an IL-12 dependent manner (59). Therefore, gut microbiota may have important implications for the immune response to ICIs.

### Liquid Biopsy

#### Circulating Tumor DNA (ctDNA)

Tumor-derived, fragmented DNA in blood is known as ctDNA, and its precise mechanism of release remains unclear but it has been postulated that it involves a passive release from dying cells and active release from living cells (137–139). It is associated with tumor burden (140), and high levels of ctDNA are an indicator of poor prognoses in patients with various types of cancer (141). Lee et al. demonstrated that melanoma patients with a persistently elevated cDNA during the treatment of anti-PD-1 antibodies show a worse response and shorter PFS and OS (60). In addition, it has been reported that, of nine melanoma patients treated with anti-PD-1 antibodies who showed pseudoprogression (defined as a tumor size increase prior to response often seen in ICI treatment), all patients had a favorable ctDNA profile defined by undetectable ctDNA at baseline or detactable ctDNA at baseline followed by >10-fold decreases (61). In contrast, in 20 patients with true progression, all but two had an unfavorable ctDNA profile defined by detectable ctDNA at baseline that remained stable or increased. These results indicate that ctDNA is a useful marker for differentiating pseudoprogression from true progression during ICI treatment.

In addition, the mutation burden of ctDNA has been also assessed and, in line with the correlation of a high mutation burden in tumor tissues, hyper-mutated ctDNA has also been associated with improved OS in patients with diverse cancers who received ICIs (142).

### Exosomes

Exosomes are microvesicles actively released from various cells, including cancer cells, and contain proteins, RNA and DNA (63). Exosomes isolated from the plasma of cancer patients contains various immune-related proteins, including PD-1, PD-L1, and CTLA-4, with PD-L1 in exosomes showing a suppressive effect on T cell activities by signaling via PD-1 (62, 143). Similar to the correlation between circulating sPD-L1 and response to ICIs, lower baseline levels, as well as increases, in exosomal PD-L1 protein have been correlated with response to pembrolizumab in melanoma patients (62). However, opposite results were observed in the association of exosomal PD-L1 mRNA expression with response to anti-PD-1 antibodies in patients with melanoma or NSCLS (63). Baseline exosomal PD-L1 mRNA expression was higher in responders compared to non-responders and exosomal PD-L1 mRNA expression in responders was significantly decreased after treatment whereas it was stable in stabilized patients and significantly increased in progressive disease cases (63). Therefore, although the mechanism is unknown, PD-L1 proteins and transcript in the exosome may provide conflicting information on ICI response.

As for other molecules, Tucci et al. recently evaluated the circulating exosomal proteins in T cells and dendritic cells in melanoma patients treated with ipilimumab (64). They demonstrated that increased exosomal PD-1 and CD28 levels in T cells were significantly associated with longer PFS and OS while increased exosomal CD80 and CD86 in dendritic cells correlated with longer PFS (64). Such exosomal proteins may reflect potential T cell/dendritic cell activities and thus lead to predictions of ICI response.

### irAE Development

Since ICIs may cause both irAEs and tumor regression through an augmented immune response, several reports have shown associations between the two events. Overall irAEs have been associated with regression of metastatic renal cell carcinoma or melanoma treated with ipilimumab (65, 144). In addition, the presence of overall irAEs was significantly associated with longer OS in melanoma patients treated with nivolumab (145). And moreover, early development of all irAEs has been associated with better ORR and longer PFS in NSCLC patients treated with nivolumab (66). However, other studies failed to show such correlations (67, 68, 146). A multivariate analysis conducted by Judo et al showed that only low grade irAEs, but not high grade irAEs, are associated with better responses to anti-PD-1 antibodies in non-melanoma patients (67). Therefore, only certain irAEs might be associated with tumor regression by ICIs. As for irAEs in each organ, several reports showed correlations between endocrine irAEs and better prognoses. Fujisawa et al. demonstrated that occurrences of endocrine irAEs were associated with longer OS in melanoma patients treated with ipilimumab after nivolumab (68). Similarly, an adjusted analysis by Kim et al. showed that development of thyroid dysfunction was significantly associated with longer PFS and OS in NSCLC patients treated with anti-PD-1 antibodies (147), suggesting that endocrine irAEs may be representative of the potential immune reaction to tumor cells. In a similar fashion, multiple studies showed that development of vitiligo correlated with better responses to ICIs in melanoma patients; this may represent a common immune response against antigens shared by melanocytes and melanomas (69, 148). Although ICIs may cause vitiligo in patients with other cancer such as NSCLC and renal cell carcinoma (149, 150), associations with outcomes in such cases remain unclear. Several studies showed that skin irAEs, except for vitiligo, were also associated with better outcome in various types of cancer (145, 148). However, Fujisawa et al. reported conflicted findings that occurrences of skin irAEs, excluding vitiligo, correlate with a shorter OS in melanoma patients treated with ipilimumab after nivolumab (68). Since skin irAEs include various types of skin disorders, such as prurigo-like eruptions, psoriasiform dermatitis and lichenoid reactions, associations with outcomes may be different for each skin irAE.

### Biomarkers of irAEs (Table 2)

The aforementioned irAEs can be induced by all ICIs. However, among the ICIs, both the frequency and the severity are highest in treatment with ipilimumab (161). Severe irAEs (grade ≥3) have occurred in 28–56% and 21–32% in patients treated with ipilimumab or anti-PD-1/anti-PD-L1 antibodies, respectively (10, 12, 16, 162, 163). In the combined treatment of ipilimumab plus nivolumab, much higher rates of severe irAEs are observed (16, 164). The organ most affected by irAEs is the skin followed by the gastrointestinal tract, respiratory tract and endocrine organs. A recent meta-analysis revealed that colitis, hypophysitis and rash were more frequent with anti-CTLA-4 antibodies whereas pneumonitis, hypothyroidism, arthralgia, and vitiligo were more common with anti-PD-1 antibodies (165). Most of these irAEs occur within 3–6 months from the initiation of ICI treatment (166–168). Given that most are mild and reversible if they are detected early and properly managed, biomarkers for predicting the occurrence of irAEs are essential. Compared with biomarkers for tumor response, those for irAEs have been less thoroughly investigated and some of the reported biomarkers for irAE overlap with those for tumor responses.

**Table 2:** Biomarkers for irAEs.

**Biomarkers**	**Cancer type**	**Patient number**	**Treatment**	**Key data and clinical significance**	**References**	**Evidence level**
Body composition parameters	Melanoma	84	Ipilimumab	Both sarcopenia and low MA were independent factors associated with high-grade irAEs.	Daly et al. (151)	2b
Sex	Melanoma	140	Ipilimumab	Females were associated with higher rates of irAEs.	Valpoine et al. (152)	2b
IL-6				IL-6 at baseline was negatively associated with irAE.		
	Melanoma	26	Ipilimumab	Lower circulating IL-6 was significantly correlated with higher incidences of colitis-related irAEs.	Chaput et al. (57)	2b
	Melanoma	15	Nivolumab	Increases in circulating IL-6 after treatment were significantly associated with development of irAEs.	Tanaka et al. (37)	4
IL-17	Melanoma	35	Ipilimumab	Circulating IL-17 levels at baseline correlated with the incidence of grade 3 irAEs of diarrhea/colitis, indicating that increased levels of circulating IL-17 may be reflective of patients with subclinical colitis.	Tarhini et al. (153)	2b
Soluble CD163, CXCL5	Melanoma	46	Nivolumab	The absolute change rate of soluble CD163 and CXCL5 after initial treatment was increased in patients with irAEs compared to those without irAEs.	Fujimura et al. (154)	2b
Blood cell counts	Melanoma, RCC, urothelial carcinom	167	Anti-PD-1 antibodies	Absolute lymphocyte and eosinophil numbers at baseline and 1 month after initial treatment were independent factors associated with a higher incidence of irAEs of grade ≥2.	Diehi et al. (155)	2b
	Melanoma	44	Anti-PD-1 antibodies	Both baseline absolute eosinophil count and relative eosinophil count at 1 month significantly correlate with the occurrence of endocrine irAEs.	Nakamura et al. (45)	2b
	Melanoma	101	Nivolumab	An increase in total WBC count and a decrease in relative lymphocyte count plus increase in relative neutrophil count on the same day of, or just prior to irAE occurrence were associated with development of lung or gastrointestinal irAEs.	Fujisawa et al. (156)	2b
autoantibodies	Melanoma, NSCLC	168	Nivolumab	TSH and TPOAb were associated with higher incidence of thyroid irAEs.	Kimbara et al. (157)	2b
	Solid cancer including melanoma, NSCLC, RCC	27	Anti-PD-1 antibodies, atezolizumab	Patients positive for type 1 diabetes antibodies at the time of presentation developed diabetes-related irAEs after fewer cycles than those without autoantibodies.	Stamatouli et al. (158)	2b
T cell repertoire	Prostate cancer	42	Ipilimumab plus granulocyte-monocyte colony-stimulating factor	An early increase in diversity and the generation of new T- cell clones correlated with the development of irAEs.	Oh et al. (159)	2b
Gut microbiome	Melanoma	26	Ipilimumab	Patients whose baseline microbiota was enriched with the *Faecalibaterium* genus and other Firmicutes showed a higher incidence of colitis-related irAEs.	Chaput et al. (57)	2b
	Melanoma	34	Ipilimumab	Increased representation of bacteria belonging to the *Bacteroidetes phylum* was associated with resistance to development of ipilimumab-induced colitis.	Dubin et al. (160)	2b

*NSCLC, non-small cell lung cancer; RCC, renal cell carcinoma; irAE, immune-related adverse event; WBC, white blood cell; TPOAb, antithyroid peroxidase antibodies (TPOAb). Evidence level was evaluated based on the following criteria; 1a, systematic review/ meta-analysis of randomized controlled trials; 1b, individual randomized controlled trials; 2a, systematic review/meta-analysis of cohort studies; 2b, individual cohort study; 3a, systematic review/meta-analysis of case-control studies; 3b, individual case-control studies; 4, case series; 5, expert opinions*.

### Body Composition Parameters

Previous reports revealed that sarcopenia was associated with poorer treatment tolerance and increased likelihood of adverse events by various chemotherapies (169, 170). In addition, low muscle attenuation (MA), which refers to increased intramuscular adipose tissue, has been associated with shorter survival in a wide variety of cancers such as melanoma and renal cell carcinoma (171, 172). Daly et al evaluated association of these body composition parameters by computer tomography with occurrences of irAEs in melanoma patients treated with ipilimumab. The multivariate analysis in this study showed that both sarcopenia and low MA were independent factors significantly associated with high-grade irAEs (151). Although the exact mechanism is unknown, many studies suggest that sarcopenia and low MA increase susceptibility to systemic inflammation (173, 174), and this may play a role in the higher frequency of severe irAEs.

### Sex

Although males have been associated with a more favorable response to ICIs, a study in melanoma patients treated with ipilimumab by Valpione et al reported that females were associated with higher rates of irAEs (152). Sex-specific factors, including hormones, play important roles in the immune response, and it is well-known that females are at a higher risk of several autoimmune diseases (175). Therefore, immune reactions to self-tissues mediated by female-specific factors may lead to an increased likelihood of irAEs.

### Serum Factors

#### IL-6

Similar to the correlation with poor tumor response, it has been reported that circulating IL-6 at baseline was negatively associated with irAE occurrence in melanoma patients treated with ipilimumab (152). Another study showed that lower circulating IL-6, as well as IL-8, was significantly correlated with higher incidences of colitis-related irAEs (57). This may be explained by the immunosuppressive effects of IL-6 in certain conditions, including the induction of MDSC (176–178). In contrast, Tanaka et al. assessed the fluctuation of multiple cytokines in melanoma patients treated with nivolumab and showed that increases in circulating IL-6 after treatment were significantly associated with development of irAEs (37). These results indicate that both lower baseline IL-6 and increase after ICI treatment may serve as predictive markers for irAE occurrence.

#### IL-17

IL-17 is a cytokine with a variety of inflammatory effects, including the recruitment of neutrophils, and it is well-known that circulating IL-17 levels are increased in patients with inflammatory bowel disease (179). Tarhini et al. assessed candidate circulating factors which were associated with irAEs in melanoma patients treated with ipilimumab as a neoadjuvant therapy and revealed that circulating IL-17 levels at baseline significantly correlated with the incidence of grade 3 irAEs of diarrhea/colitis (153). This indicates that increased levels of circulating IL-17 may be reflective of patients with subclinical colitis, the development of which would be normally inhibited by CTLA-4.

### Soluble CD163 (sCD163) and CXCL5

Circulating levels of sCD163, which is derived from macrophages, increase in various autoimmune disorders, including rheumatoid arthritis and pemphigus vulgaris, and are reflective of their activities (176, 180). CXCL5 is a chemokine which can attract CXCR2^+^ myeloid cells and can be produced by CD163^+^ macrophages. It is also known to be a biomarker for several autoimmune disorders (176, 181). Fujimura et al evaluated circulating sCD163 and CXCL5 levels at baseline and day 42 after initial treatment with nivolumab in melanoma patients (154), showing that the sCD163 absolute change rate was significantly increased in patients with irAEs compared to those without irAEs. Although there were no significant differences, the absolute change rate of CXCL5 also tended to be higher in patients with irAEs, suggesting that absolute changes within sCD163 and CXCL5 levels after ICI treatment could serve as possible biomarkers for irAE development.

### Blood Cells

Since both T cells and eosinophils are crucial for cellular immunity, blood cell counts of these cells may also be correlated with irAE development. A multivariate analysis conducted by Diehi et al. demonstrated that, in solid tumor patients (including melanoma, renal cell carcinoma, and urothelial carcinoma) treated with anti-PD-1 antibodies, absolute lymphocyte and eosinophil numbers at baseline and 1 month after initial treatment were independent factors that were significantly associated with a higher incidence of irAEs of grade ≥2 (155). In addition, our study demonstrated that both baseline absolute eosinophil count and relative eosinophil count at 1 month significantly correlate with the occurrence of endocrine irAEs in melanoma patients treated with anti-PD-1 antibodies (45). Therefore, circulating lymphocyte and eosinophil numbers may predict not only tumor responses but also the occurrence of ICI-mediated irAEs.

In contrast, Fujisawa et al. investigated fluctuations in blood cell count on the same day of, or just prior to irAE occurrence, in melanoma patients treated with nivolumab (156). Univariate analyses revealed that increases in total white blood cell (WBC) count and decreases in relative lymphocyte count from baseline were associated with severe irAEs of grade ≥3 although multivariate analyses failed to show independence. They also analyzed the correlation with irAEs of each organ and found that the same factors, namely an increase in total WBC count and a decrease in relative lymphocyte count plus increase in relative neutrophil count, were significantly associated with development of lung or gastrointestinal irAEs. This could be caused by neutrophil-dominant infiltration into the affected organs since DAMPs from severely damaged cells promote neutrophil recruitment (182). Indeed, active colitis in patients treated with ipilimumab saw severe neutrophil infiltration into the lamina propria (183), indicating that these factors may be useful for predicting irAEs that are currently developing or may soon develop.

### Autoantibodies

Detection of autoantibodies is speculated to predict development of irAEs related to the autoantibodies (184). Kimbara et al. assessed TSH, free T3, free T4, antithyroid peroxidase antibodies (TPOAb) and antithyroglobulin antibodies at baseline in patients with solid tumors treated with nivolumab and multivariate analyses revealed that TSH and TPOAb were significantly associated with higher incidence of thyroid irAEs (157). Stamatouli et al. measured diabetes autoantibodies (glutamic and decarboxylase 65 antibodies, islet antigen 2 antibodies, and insulin autoantibodies) in solid cancer patients treated with anti-PD-1 or anti-PD-L1 antibodies, and found that patients positive for type 1 diabetes antibodies at the time of presentation developed diabetes-related irAEs after fewer cycles than those without autoantibodies (158). They also measured autoantibodies prior to treatment in three patients, and one was already positive, indicating that autoantibodies may be useful to predict their related irAEs.

### T Cell Repertoire

The T cell repertoire has been reported to correlate with irAEs as well as tumor response. Oh et al assessed the repertoire of circulating T cells in patients with metastatic castration-resistant prostate cancer treated with a combination of ipilimumab and granulocyte-monocyte colony-stimulating factor (159). They found that initial broadening in the repertoire occurred within 2 weeks of treatment, which significantly preceded irAEs onset, and an early increase in diversity and the generation of new clones were correlated with the development of irAEs. These results suggest that increased T cell diversity in response to ICI treatment could be a sign of immune response to normal tissues as well as tumor tissues.

### Gut Microbiome

It is suggested that inflammatory bowel diseases (IBD) may result from a loss of tolerance to commensal bacteria and dysbiosis is a well-known factor that is significantly involved in the pathogenesis of IBD (185). Gut microbiota have been also reported as predictive of colitis-related irAEs. Melanoma patients treated with ipilimumab whose baseline microbiota was enriched with the *Faecalibaterium* genus and other Firmicutes showed a higher incidence of colitis-related irAEs although they were also associated with better outcomes (57). In contrast, this study showed no occurrences of colitis irAEs in any patients with *Bacteroidetes* (57). Similarly, Dubin et al. demonstrated that increased representation of bacteria belonging to the *Bacteroidetes phylum* was associated with resistance to development of ipilimumab-induced colitis (160).

### Tumor Type

A recent meta-analysis demonstrated that the frequency of each type of irAE depends on cancer type (165). Melanoma patients had a higher frequency of skin and gastrointestinal irAEs but a lower frequency of pneumonia compared with NSCLC patients (165). In addition, dermatitis, arthritis and myalgia were more frequent in melanoma patients than in renal cell carcinoma patients whereas pneumonitis and dyspnea were found to be less common in melanoma cases (165). Although the precise mechanism remains unclear, induced immune responses to antigens of normal tissue shared with or cross-reactive with those of each cancer may be an explanation.

## Conclusion

Although numerous predictive biomarkers for tumor response and irAEs during ICI treatment have been identified, there are no absolutely predictive biomarkers as yet. Therefore, multiple biomarkers should be taken into consideration in choosing or quitting ICI treatments. Because immune reactions induced by ICIs are quite complex and many factors are involved, identifying new biomarkers will provide mechanistic insights into the ways how ICIs modulate the anti-tumor response and irAEs in specific patients, as well as lead to the development of novel treatments to target the identified biomarkers.

## Author Contributions

The author confirms being the sole contributor of this work and has approved it for publication.

### Conflict of Interest Statement

The author declares that the research was conducted in the absence of any commercial or financial relationships that could be construed as a potential conflict of interest.
